# Ultrasound Echogenicity as an Indicator of Muscle Fatigue during Functional Electrical Stimulation

**DOI:** 10.3390/s22010335

**Published:** 2022-01-03

**Authors:** Qiang Zhang, Ashwin Iyer, Krysten Lambeth, Kang Kim, Nitin Sharma

**Affiliations:** 1 UNC/NCSU Joint Department of Biomedical Engineering, North Carolina State University, Raleigh, NC 27695, USA; qzhang25@ncsu.edu (Q.Z.); aiyer3@ncsu.edu (A.I.); kflambet@ncsu.edu (K.L.); 2 UNC/NCSU Joint Department of Biomedical Engineering, The University of North Carolina at Chapel Hill, Chapel Hill, NC 27514, USA; 3 The Department of Bioengineering, School of Engineering, University of Pittsburgh, Pittsburgh, PA 15260, USA; kangkim@upmc.edu; 4 The Center for Ultrasound Molecular Imaging and Therapeutics, Department of Medicine and Heart and Vascular Institute, University of Pittsburgh School of Medicine, University of Pittsburgh Medical Center, Pittsburgh, PA 15213, USA; 5 The Department of Mechanical Engineering and Materials Science, School of Engineering, University of Pittsburgh, Pittsburgh, PA 15260, USA; 6 The McGowan Institute for Regenerative Medicine, University of Pittsburgh Medical Center, University of Pittsburgh, Pittsburgh, PA 15219, USA

**Keywords:** muscle fatigue, electrical stimulation, ankle joint, biomechanical phenomena, ultrasonography, linear models, nonlinear dynamics

## Abstract

Functional electrical stimulation (FES) is a potential neurorehabilitative intervention to enable functional movements in persons with neurological conditions that cause mobility impairments. However, the quick onset of muscle fatigue during FES is a significant challenge for sustaining the desired functional movements for more extended periods. Therefore, a considerable interest still exists in the development of sensing techniques that reliably measure FES-induced muscle fatigue. This study proposes to use ultrasound (US) imaging-derived echogenicity signal as an indicator of FES-induced muscle fatigue. We hypothesized that the US-derived echogenicity signal is sensitive to FES-induced muscle fatigue under isometric and dynamic muscle contraction conditions. Eight non-disabled participants participated in the experiments, where FES electrodes were applied on their tibialis anterior (TA) muscles. During a fatigue protocol under either isometric and dynamic ankle dorsiflexion conditions, we synchronously collected the isometric dorsiflexion torque or dynamic dorsiflexion angle on the ankle joint, US echogenicity signals from TA muscle, and the applied stimulation intensity. The experimental results showed an exponential reduction in the US echogenicity relative change (ERC) as the fatigue progressed under the isometric ($R^{2}=0.891\pm 0.081$) and dynamic ($R^{2}=0.858\pm 0.065$) conditions. The experimental results also implied a strong linear relationship between US ERC and TA muscle fatigue benchmark (dorsiflexion torque or angle amplitude), with $R^{2}$ values of 0.840±0.054 and 0.794±0.065 under isometric and dynamic conditions, respectively. The findings in this study indicate that the US echogenicity signal is a computationally efficient signal that strongly represents FES-induced muscle fatigue. Its potential real-time implementation to detect fatigue can facilitate an FES closed-loop controller design that considers the FES-induced muscle fatigue.

## 1. Introduction

(Note that preliminary results have been published in [1]; however, those results employed US echogenicity post-processing as an offline manner and contained experimental data from only three participants.) Neurological injuries, like a spinal cord injury (SCI) [2] and stroke [3], usually result in paraplegia or hemiplegia, disrupting both physical and emotional well-beings [4]. Without physical assistance from mobility aids or a neuroprosthetic intervention, the mobility impairment increases social isolation, anxiety, and depression. Functional electrical stimulation (FES), an artificial technique that applies low-amplitude electrical potentials across the paralyzed skeletal muscle belly or peripheral nerve, can reanimate the walking function and help restore mobility. Since the earlier demonstrations of FES to correct drop foot [5,6], recent studies [7,8,9,10,11,12] investigated its orthotic effects on a larger clinical population. Additionally, FES can provide supplementary benefits, including the improvement in muscle tone and size, muscle strength, blood flow, and a reduction in muscle spasticity and disuse osteoporosis. Despite the efficacy and benefits of FES, the rapid onset of muscle fatigue is a major limitation. Due to the non-selective stimulation nature of FES, peripheral motor units are synchronously activated and discharged, causing the stimulated muscle to fatigue easily. The induced fatigue results in the deterioration of the muscle contraction force generation, causing a rapid loss of FES control effectiveness [13].

To reduce the FES-induced muscle fatigue, multiple studies have investigated the spatially distributed sequential stimulation pattern [14,15,16], where a single stimulation site distributes the center of the electrical field over a wide area by using an array of surface electrodes. In addition, Downey et al. [17] showed that the use of multi-channel asynchronous stimulation reduced muscle fatigue compared to conventional single-channel stimulation. Later in [18], a closed-loop controller for asynchronous FES was shown to extend the duration of functional movements. Despite the advances in stimulation protocols and new closed-loop FES controllers, non-invasive evaluation and characterization of the FES-induced muscle fatigue are lacking. Fatigue measurement methods are important for quantifying the fatigue effects on the neuromusculoskeletal dynamics and for an effective FES control design.

Efforts in indirectly measuring fatigue include, but are not limited to, tetanic contraction force measurement [19], electromyography (EMG)/surface electromyography (sEMG) [20,21,22], mechanomyography [23], near-infrared spectroscopy [24,25,26], and phosphorus nuclear magnetic resonance [27]. Among these technologies, sEMG is the most well-developed and convenient non-invasive methodology to assess peripheral muscle fatigue. Although [28,29,30] report successful extraction of volitional or evoked sEMG during FES, the analysis and evaluation of the EMG signals during FES is still challenging. The challenges are mainly due to the FES-induced contractions cluttering and masking the pure sEMG signals [31,32], interference and cross talk from adjacent muscles [21], and the inability to measure the sEMG signals from deeply seated muscles [33]. Recently, ultrasound (US) imaging technique, know as sonomyography, has been investigated to qualitative or quantitatively assess muscle fatigue for volitional and FES-induced muscle contraction as an alternative methodology to sEMG. Due to a relatively high spatial and temporal resolution, the US images provide direct visualization of the muscle deformations during the implementation of FES. These muscle deformations can be quantified to obtain a comprehensive measure that reflects the fatigue effect. Shi et al. [34] used muscle thickness, extracted from cross-sectional US images, to characterize the volitionally induced fatigue in the biceps brachii muscles. Similarly, Witte et al. [35] applied US strain imaging to capture the elastic and viscoelastic-like modifications in the 3rd flexor digitorum superficialis muscle after a voluntary fatiguing exercise. Sheng et al. [36,37] investigated an adaptive speckle tracking algorithm for determining strain changes in the quadriceps muscle during the FES-induced muscle fatigue protocol under isometric knee extensions.

The aforementioned US imaging-related studies for assessing muscle fatigue primarily investigated isometric muscle contractions. Few studies have investigated FES-induced muscle fatigue characteristics under dynamic joint movement conditions. Additionally, the aforementioned studies reported their results based on offline processed US imaging data, since deriving fatigue-relevant features from US imaging is generally computationally intensive. The high computation cost significantly limits the use of US imaging to evaluate muscle fatigue in real-time.

Inspired by recent studies in US imaging-derived echogenicity signals to predict motion intent or voluntary effort in the forearm and ankle muscles [38,39], preliminary results of using post-processed US echogenicity signal to assess the FES-induced muscle fatigue have been reported in [1]. In this work, we extended the preliminary results in [1] to a larger participant group and investigated the feasibility of using the online-processed US echogenicity to quantitatively assess FES-induced muscle fatigue. Specifically, the tibialis anterior (TA) muscle was selected to reveal the fatigue-indicating performance of US echogenicity under both isometric and dynamic ankle dorsiflexion movements, where we synchronously collected dorsiflexion force/angular position (isometric/dynamic conditions), TA muscle’s US echogenicity, and FES intensity during the muscle fatigue progression. A comprehensive correlation analysis between the temporal US echogenicity relative change (ERC) and TA muscle fatigue progression (decay of dorsiflexion force or angle during isometric or dynamic conditions) was performed to assess the muscle contractility during fatigue progression. It was hypothesized that there exists a nonlinear relationship between the US ERC and the FES duration (contraction cycles), as well as a linear relationship between the US ERC and the decay of dorsiflexion force or angle. Furthermore, the performance of US ERC as a surrogate metric of muscle fatigue was compared to the US tissue strain as reported in [36,37].

## 2. Materials and Methods

### 2.1. Subjects

The study was approved by the Institutional Review Board (IRB) at North Carolina State University (IRB approval number: 20602). The study is also in accordance with the ethical standards of the Helsinki Declaration. Eight participants without any neuromuscular disorders (age: 26.0 ± 2.2 years, height: 173.7 ± 5.9 cm, weight: 72.6 ± 11.2 kg, 3F/5M) were recruited to complete FES-elicited ankle dorsiflexion experiments during isometric and dynamic conditions. Every participant signed an informed consent form before taking part in the experiments. The participants were identified as Sub01, Sub02, …, Sub08. The current study was a pilot-designed study or a proof-of-concept study with a relatively small sample size. We applied an a priori sample size estimation before the experiments. The hypotheses of this paper are that there exists a nonlinear relationship between the US ERC and the FES duration, as well as a linear relationship between the US ERC and the decay of dorsiflexion torque/angle. So, the null hypotheses would be that no obvious nonlinear or linear relationships exist between the US ERC and the FES duration or between the US ERC and the decay of dorsiflexion torque/angle. To control the risk of accepting a false hypothesis, the probability of rejecting the null hypothesis when it is true, α, was set as 0.05, and the probability of accepting the null hypothesis when it is wrong, β, was set as 0.10. Then the minimum sample size *N*, is calculated below for the one-sided test of hypothesis with standard deviation *s* assumed to be known and equal to the shift δ.

(1) $$N={\left(t_{1-\alpha /2}+t_{1-\beta }\right)}^{2}{\left(\frac{s}{\delta }\right)}^{2}={\left(1.64+1.28\right)}^{2}\approx 8.$$

### 2.2. Experimental Protocol and Data Collection

**Figure 1:**
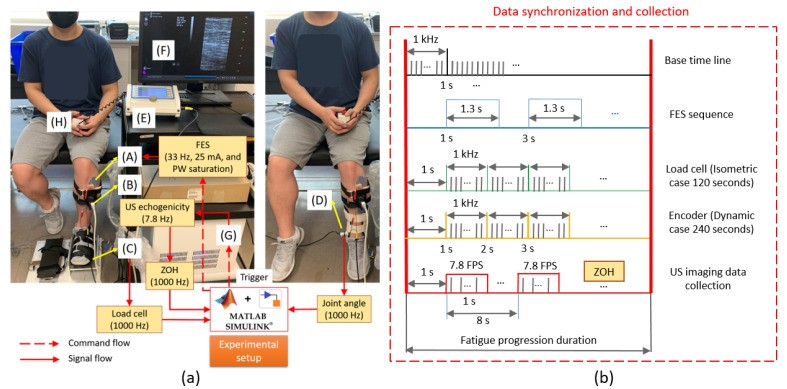
(**a**) Experimental setup of the isometric (left) and dynamic (right) ankle joint dorsiflexion by using FES. A—FES electrode pads, B—Prodigy US transducer, C—Load cell platform, D—Incremental encoder, E—FES stimulator, F—Monitor showing B-mode US imaging, G—Prodigy US machine, H—Safety stop button. (**b**) Data synchronization and collection among multiple channels.

The isometric and dynamic experimental setup is illustrated in Figure 1a. Detailed descriptions of the isometric setup, including the load cell platform (C) and US imaging transducer (B) and processing machine (G), can be found in [39,40]. In the dynamic experimental setup, the participant’s foot was suspended to ensure the full range of motion for both dorsiflexion and plantarflexion. A wearable ankle brace connected with an incremental encoder (D) (1024 pulses per revolution, TRD-MX1024BD, AutomationDirect, GA, USA) was inserted into the participant’s shoes, and two pieces of free movable components were stamped to the shank. Thus, the ankle motion was constrained in the sagittal plane and measured by the encoder. The seated posture in Figure 1a was maintained throughout isometric and dynamic experimental procedures. Two electrodes (A) (size: 2${}^{\prime }$${}^{\prime }$× 2${}^{\prime }$${}^{\prime }$) were placed on the fibular head and the distal belly of the TA muscle, respectively. The electrodes applied bi-phasic stimulation pulse trains from a commercial stimulator (E) (Rehastim 2, HASOMED GmbH, Magdeburg, Germany). A region approximately 30% to 50% of individual shank length distal from the rotation central line of the knee joint was chosen as the location for the US transducer placement. The depth of US imaging was set at 40 mm to include the entire TA muscle area. A monitoring screen (F) that displayed B-mode US images was used to adjust the US transducer location and orientation to guarantee good visualization and resolution of the TA muscle.

There were three separate experimental tasks performed on three different days. At least 72 h were provided for two successive tasks to ensure a full recovery and mitigate muscle fatigue effects from the last task. For each experimental task, participants were instructed to avoid any volitional TA muscle contraction. Throughout those three experimental tasks, the FES current amplitude was set as 25 mA and the stimulation frequency as 33 Hz for all participants. The first task was conducted under the isometric condition to determine subject-specific FES pulse width threshold and saturation values, following the procedures described in [41]. The second and third sets were conducted randomly over two days to analyze muscle fatigue in both isometric and dynamic conditions. The threshold pulse width amplitude of each individual was taken as the amplitude that produced the first significant increase of the dorsiflexion torque. The pulse width saturation was taken as the amplitude that no longer generated a significant increase in the dorsiflexion torque. During the first task, the pulse width was increased from 0 μs to 600 μs with an increment of 20 μs and with an activation period of 1 s every 5 s. After the personalized pulse width saturation was determined from the first experimental task, 80% of individual pulse width saturation was applied for the second and third tasks to facilitate the isometric and dynamic fatigue protocols. Figure 1b presents the protocol for FES-induced TA muscle fatigue progression, data synchronization, and collection. The first second was left blank to initialize all channels for data collection. A time base of 1000 Hz was run in a Simulink (R2019b, MathWorks Inc., Natick, MA, USA) real-time program on a target machine (Speedgoat Inc., Liebefeld, Switzerland).

The participants felt stronger muscle deformations under the isometric condition than the dynamic condition, even with the same stimulation intensity. Therefore, the time periods of those two fatigue progressions were set differently. With respect to the aforementioned time base, two fatigue progression periods of 120 s and 240 s were applied for the isometric and dynamic conditions, respectively. FES was activated every 2 s with a duty cycle of 65%. Under the isometric and dynamic fatigue progressions, the dorsiflexion force signal and dorsiflexion angle signal were collected at 1000 Hz throughout the entire period.

The US echogenicity signal was acquired offline in our previous studies [1,39], where the plane-wave US imaging radio frequency (RF) data were collected and saved on the US machine at a frame rate of 1000 frames per second. A delay-and-sum beamforming method was applied offline to generate the US image time sequence and the US echogenicity signal time sequences were calculated post hoc. The RF data collection was triggered by signals from the target machine for synchronizing with the collection of dorsiflexion torque or dorsiflexion angle. Although that approach makes it extremely suitable for applications where fast phenomena and tiny deformations need to be observed, this plane wave imaging at high temporal resolution (1000 Hz) significantly degrades the spatial resolution (image quality), thus resulting in more noise when calculating US echogenicity. In addition, the offline beamforming and echogenicity calculation are not feasible for FES closed-loop control with the US imaging-derived signal as feedback in real-time.

In the current study, we developed and implemented the online US image beamforming and echogenicity calculations on the US machine according to the series steps of “line-by-line beamforming—image cropping—US echogenicity calculation—data transfer to Simulink”, which required a lot of computational capability and time to make this online US echogenicity stream available. During the muscle fatigue progression, while the US echogenicity transmission was running online, raw RF data were also saved for US imaging visualization in a post hoc way. To reduce the computation and data storage burden of the US machine, during both isometric and dynamic fatigue progressions, US echogenicity signals and raw RF data were collected synchronously with signals from the load cell or the encoder during the first second of every 4 stimulation cycles, as illustrated in Figure 1b. Preliminary results showed the above online steps could run around 7.8 times per second, so the online US echogenicity was sent out from the US machine at a frequency of 7.8 Hz. Due to the use of a zero-order-hold function in Simulink, the US echogenicity data collection was still sampled and collected at 1000 Hz, but without changes during two successively generated values from the US machine. The details of the US echogenicity calculation are explained in the following subsection. The aforementioned experimental data collection procedures were applied on the left ankle joint of each participant.

### 2.3. Data Processing and Analysis

**Figure 2:**
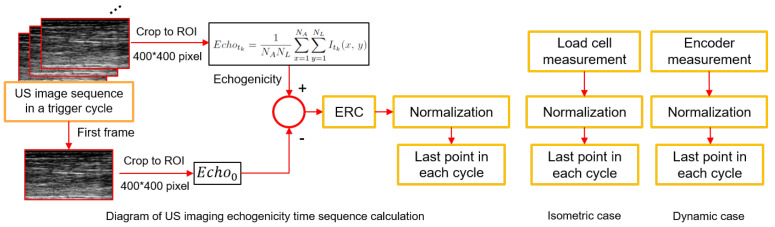
Diagram of data processing, including US imaging echogenicity, load cell, and encoder measurements under both isometric and dynamic conditions.

The ankle dorsiflexion torque and angle measurements were low-pass filtered by a 4th-order Butterworth filter with a cutoff frequency of 6 Hz. According to the data synchronization in Figure 1b, the dorsiflexion torque signal during the isometric condition and dorsiflexion angle during the dynamic condition were aligned with the period when FES was on, and the last data point from each stimulation cycle was selected and normalized to the peak value across all stimulation cycles for further analysis. Similarly, the dorsiflexion torque or dorsiflexion angle signals were aligned with the period when the US imaging trigger was on, and the last data point from each trigger cycle was selected and normalized to the peak value across all US imaging trigger cycles for further analysis. The detailed data processing diagram is presented in Figure 2.

Here are the procedures for US imaging data processing. First, the raw RF data were beamformed online through the line-and-line beamforming algorithm. Then the logarithmic imaging intensity compression was performed to get the envelope of the demodulated RF data. By normalizing the envelope of each pixel between 0 and 255, the B-mode US image at the current frame was generated. A median filter and non-local means denoising [42] were applied to spatially filter each B-mode image. At last, the averaged gray-scaled echo intensity within the region of interest (ROI) of 400 pixel × 400 pixel was calculated as the echogenicity value for the current US image frame [38]. Therefore, the sequential US echogenicity signal was calculated as

(2) $$Echo_{t_{k}}=\frac{1}{N_{A}N_{L}}\sum_{x=1}^{N_{A}}\sum_{y=1}^{N_{L}}I_{t_{k}}\left(x,\;y\right),$$

where $N_{A},\;N_{L}$ represent the pixel numbers along with axial and lateral directions, respectively. $I_{t_{k}}\left(x,\;y\right)$ represents the US intensity information at the pixel location (x,y) on the image at $t_{k}$ instant from the normalized logarithmic imaging intensity compression signals. As a consequence, the 2D image time sequence was transferred to a 1D signal time sequence. Visually, each $I_{t_{k}}\left(x,\;y\right)$ presents the brightness of that pixel at the location of (x,y) on the 2D map. Thus, the calculated echogenicity signal presents an overall brightness within the ROI. Note that although the US imaging beamforming and echogenicity calculation were based on the online manner, the updating frequency was fairly low (7.8 Hz from preliminary results) due to the computation time and communication delay between the US machine and Simulink real-time program. Therefore, a zero-order-hold function was used in the real-time program to collect the online calculated US echogenicity at 1000 Hz. The echogenicity time sequence within the same stimulation cycle was subtracted by the echogenicity of the first image from the same cycle, which was defined as the ERC within the same cycle. Similarly, the last data point of ERC in each trigger cycle was selected and normalized to the peak ERC throughout all trigger cycles. Given the FES-induced fatigue progression protocol, the last point of ERC corresponded to a sub-maximal dorsiflexion force or dorsiflexion angle; therefore, the aligned last data point of dorsiflexion force or angle during each FES cycle and the aligned last data point of ERC during each US imaging trigger cycle were selected to characterize the muscle contractility during fatigue progressions. As a consequence, during the isometric fatigue progression, 60 samples from dorsiflexion forces and 15 samples from ERC were obtained, while during the dynamic fatigue progression, 120 samples from the dorsiflexion angles and 30 samples from ERC were obtained.

According to the muscle fatigue dynamics and its solution mentioned in [13], an exponential regression model was used to fit the curve between the normalized sub-maximal dorsiflexion force or angle and the index number of contractions (i=1,2,…,60/i=1,2,…,120), as well as the curve between the normalized sub-maximal ERC and the index number of contractions (i=4,8,…,60/i=4,8,…,120). The coefficients of the exponential regression models were determined by using the Levenberg–Marquardt nonlinear least squares algorithm [43]. A linear regression model was used to fit the line between the normalized sub-maximal dorsiflexion torque/angle and the normalized sub-maximal US ERC. To evaluate the goodness of curve fittings, the coefficient of determination ($R^{2}$) of each regression model was also calculated as

(3) $$R^{2}=\frac{{\left(\sum_{i=1}^{N}\left(T_{i}-\bar{T}\right)\left({\hat{T}}_{i}-\bar{\hat{T}}\right)\right)}^{2}}{\sum_{i=1}^{N}{\left(T_{i}-\bar{T}\right)}^{2}\sum_{i=1}^{N}{\left({\hat{T}}_{i}-\bar{\hat{T}}\right)}^{2}},$$

where $T_{i}$ and ${\hat{T}}_{i}$ denote each measured sub-maximal variable point and output from the regression model, respectively. $\bar{T}$ and $\bar{\hat{T}}$ denote the average of the measured sub-maximal variable and the average of the output from regression model, respectively.

### 2.4. Statistical Analysis

The normality of the targeted data sets was tested based on the Shapiro–Wilk parametric hypothesis test (SW test). Those data sets include coefficients and $R^{2}$ values of each exponential regression model and linear regression model under either isometric or dynamic conditions across all eight participants, as well as the computation times of the US echogenicity and axial strain per image frame. According to the results from SW test, a paired *t*-test (normal distribution) or a Wilcoxon signed rank test (not normal distribution) was applied to analyze if there was significant difference between two independent groups. To be more specific, for the exponential and linear regression models, the optimal coefficient values were compared to zero, and $R^{2}$ values were compared under isometric and dynamic conditions.

As a comparative study, the $R^{2}$ values between the normalized ERC and normalized sub-maximal torque under the isometric condition were compared to the results reported in [36] between the normalized maximal axial strain and normalized sub-maximal torque. In addition, the computation times of the US echogenicity and axial strain per image frame were also compared to determine if there was a significant difference between these two muscle fatigue indicators. For all statistical analysis, the significant difference level was set as *p* < 0.05.

## 3. Results

### 3.1. Individual FES Pulse Width Threshold and Saturation Determination

**Figure 3:**
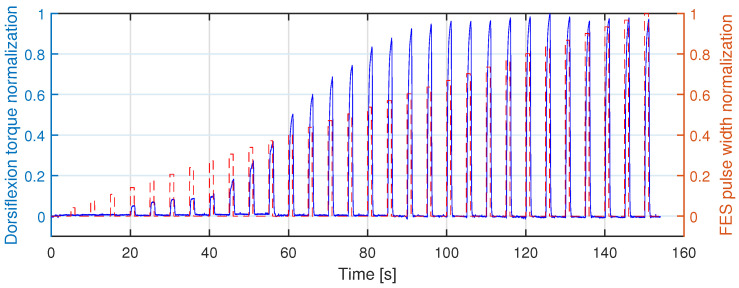
The normalization of FES pulse width that applied on the TA muscle and the normalization of ankle dorsiflexion torque measurements on Participant Sub01 during the first task.

The experimental results from the first task on Sub01 are presented in Figure 3, where the monotonically increasing FES pulse width and ankle dorsiflexion torque are normalized to their corresponding peak values during the entire task. The threshold pulse width amplitude of each individual was taken as the amplitude that produced the first significant increase of the dorsiflexion torque, while the pulse width saturation was taken as the amplitude that no longer generated a significant increase of dorsiflexion torque. According to the dorsiflexion torque increase in Figure 3, the pulse width threshold and saturation for Sub01 are around 100 μs and 420 μs, respectively. Similarly, the same determination approach was applied to all other participants, and the pulse width threshold and saturation values are summarized in Table 1.

### 3.2. TA Muscle Fatigue Effects on Isometric and Dynamic Ankle Dorsiflexion

**Figure 4:**
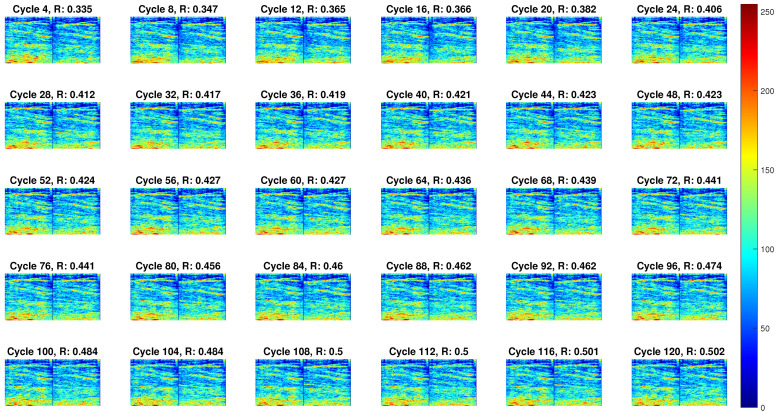
The first and last frames of US imaging from every 4 stimulation cycles under the dynamic fatigue progression on Participant Sub03.

Taking the FES-induced TA muscle fatigue under the dynamic condition on Participant Sub03 as an example, the qualitative evaluation of muscle contractility characteristics during the fatigue progression can be visualized in Figure 4. The first and last frames of US imaging from every 4 stimulation cycles were selected and compared in each subplot of Figure 4. According to the negative correlation between the echogenicity signals and muscle contraction levels in [39], the hyperechogenic (with higher gray-scaled values) and hypoechogenic (with lower gray-scaled values) US images represent less and more muscle contraction force, respectively. It is observed that with the increase of stimulation cycles, the last frame of US imaging becomes more hyperechogenic, which indicates the TA muscle force generation ability decreases. The 2D correlation coefficient between the presented two frames in each stimulation cycle was also calculated and shown in each subplot. A higher correlation coefficient represents smaller deformation of the targeted muscle, indicating less muscle contraction force generation. It is observed that the correlation coefficient increases along with the stimulation cycles, representing the reduced muscle force generation due to FES-induced muscle fatigue. A similar changing pattern of US imaging was also observed under the isometric and dynamic conditions of other participants. Representative videos displaying the temporal and spatial changes of TA muscle US imaging under both isometric and dynamic FES-induced fatigue progression on Sub03 and Sub05 are included in the Supplementary Materials.

**Figure 5:**
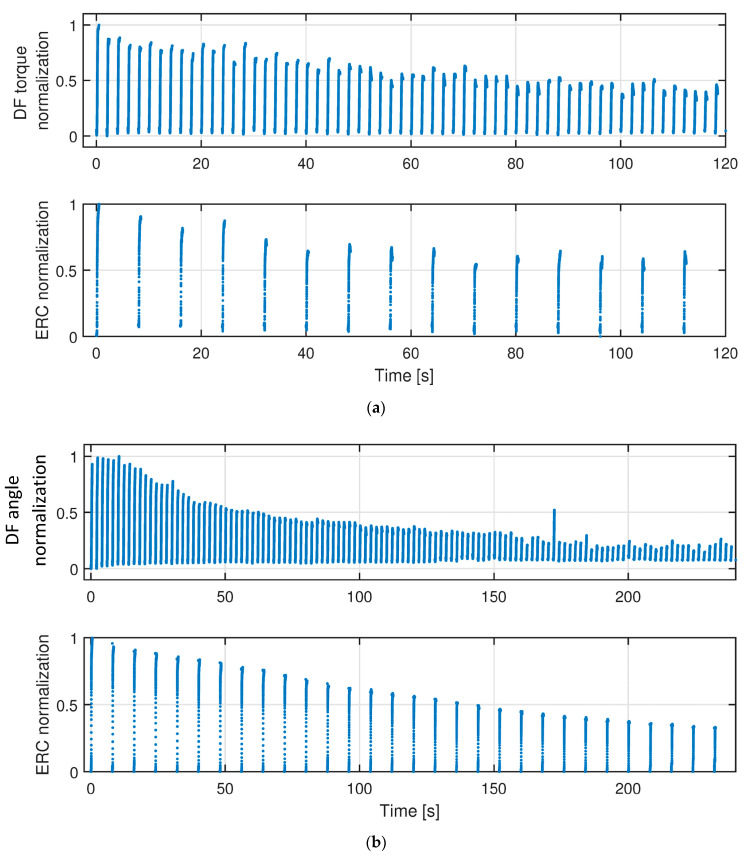
The representative effects of FES-induced TA muscle fatigue on each recorded stimulation cycle of the isometric dorsiflexion torque normalization, dynamic dorsiflexion angle normalization, and US ERC normalization on Participant Sub03. (**a**) Normalization of dorsiflexion torque and ERC in each recorded stimulation cycle due to TA muscle fatigue under the isometric condition. (**b**) Normalization of dorsiflexion angle and ERC in each recorded stimulation cycle due to TA muscle fatigue under the dynamic condition.

**Figure 6:**
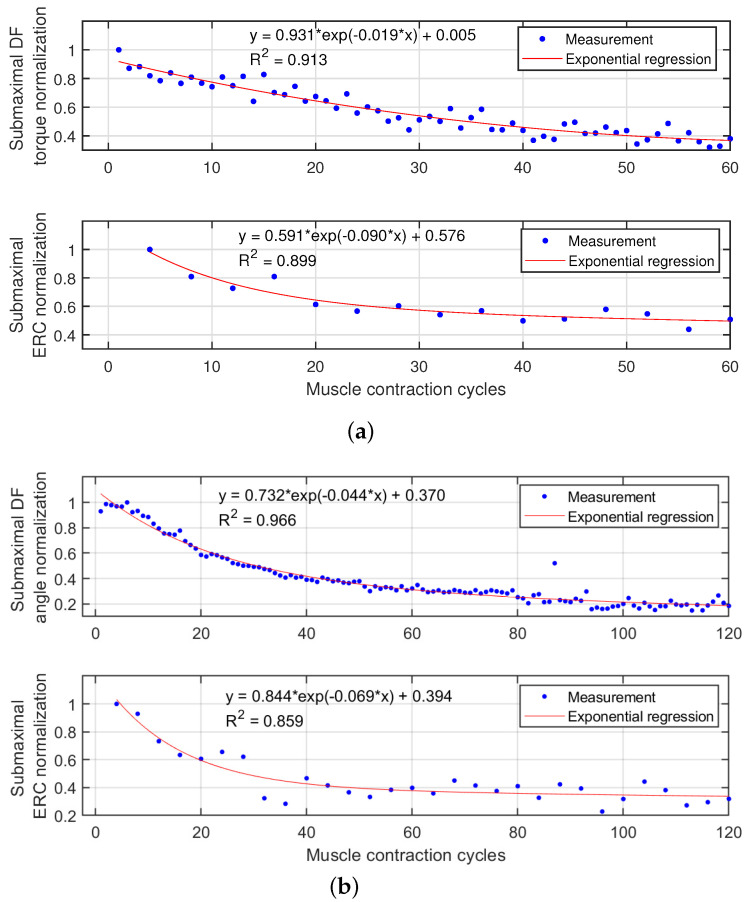
Results of the last data point of each recorded stimulation cycle, including the isometric dorsiflexion torque normalization, dynamic dorsiflexion angle normalization, and US ERC normalization on Participant Sub03. Furthermore, this figure includes the exponential regression equations and *R*${}^{2}$ values of each variable decay curve along with the muscle contraction number. (**a**) Last data point of normalized dorsiflexion torque and ERC in each recorded stimulation cycle under the isometric condition. (**b**) Last data point of normalized dorsiflexion angle and ERC in each recorded stimulation cycle under the dynamic condition.

To evaluate the FES-induced fatigue, the reduction of dorsiflexion torque or angle was considered as the benchmark. Corresponding to the benchmark, we observed the reduction of ERC during the muscle fatigue progression. The representative results of TA muscle fatigue progression from Sub03 are shown in Figure 5, where each curve on the top subplot represents dorsiflexion torque (a) or angle normalization (b) continuous change during each recorded contraction cycle. Each curve on the bottom subplot represents the corresponding ERC normalization change during the first recorded contraction cycle every 4 stimulation cycles. As mentioned in the last section, the last data point of each variable curve was selected, which represents the sub-maximal value for each variable during each recorded contraction cycle. The scattered plots between the last data point of each variable and TA muscle stimulation cycle are presented in Figure 6. Remarkably, all signals show a monotonic decay trend with the muscle fatigue progression. In Figure 6a, the sub-maximal dorsiflexion torque reduces to 50% of the pre-fatigue capability after about 35 contraction cycles, while the sub-maximal dorsiflexion angle reduces to 50% of the pre-fatigue capability after about 30 contraction cycles. Additionally, after 60 stimulation cycles, the dorsiflexion torque and angle decayed to 39.2% and 31.2% of the pre-fatigue capacity under isometric and dynamic conditions, respectively. The results indicate that, with the same FES intensity and same muscle stimulation cycles, the fatigue levels of the TA muscle are similar under isometric and dynamic conditions. However, the participants reported that they feel more comfortable during the fatigue progression under the dynamic condition. Under both conditions, as the increase of muscle contraction cycles, the isometric dorsiflexion torque and dynamic dorsiflexion angle present a strong exponential decay. The exponential regression equations and *R*^2^ values are labeled on upper plots of Figure 6a,b. On lower plots of Figure 6, although with even sparser measurement points, a strong exponential relationship is still observed between the ERC normalization and the stimulation cycles for both isometric and dynamic conditions. The exponential regression equations and $R^{2}$ values are labeled in Figure 6. For other participants, the coefficients of exponential regression models and corresponding $R^{2}$ values are listed in Table 2, where the upper (lower) half represents the regression model between dorsiflexion torque/angle normalization (ERC normalization) and contraction cycles.

**Figure 7:**
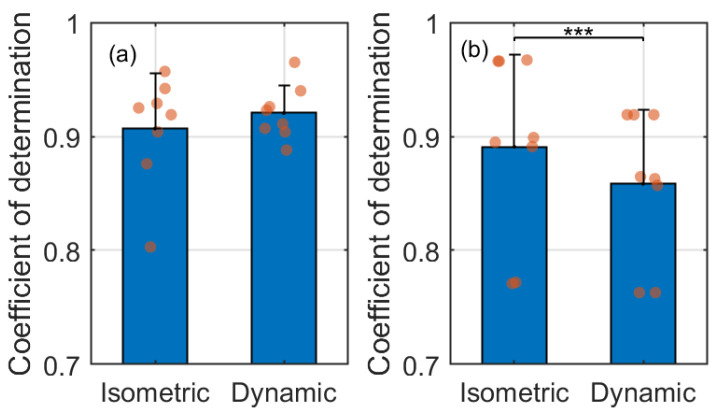
The comparison results of the coefficients of determination under isometric and dynamic conditions. (**a**) Exponential regression model between the dorsiflexion torque/angle normalization and muscle contraction cycles, (**b**) Exponential regression model between the ERC normalization and muscle contraction cycles. *** represents the significant difference level of p<0.001.

Usually, a standard criterion to evaluate the goodness of the regression performance is that the $R^{2}$ value is higher than or equal to 0.8. For the exponential regression model between the dorsiflexion torque/angle normalization and muscle contraction cycles, $R^{2}$ values are higher than 0.8 across all participants and both conditions from Table 2. For the exponential regression model between the ERC normalization and muscle contraction cycles, $R^{2}$ values are higher than 0.8 except for Sub02 and Sub05 under both conditions. Furthermore, Figure 7 shows comparison results of the $R^{2}$ values between the isometric and dynamic conditions. No significant difference is observed between $R^{2}$ values of torque-contraction cycle-regression (mean ± standard deviation: 0.907 ± 0.048) and $R^{2}$ values of angle-contraction cycle-regression (mean ± standard deviation: 0.921 ± 0.024). However, $R^{2}$ values of ERC-contraction cycle-regression during the isometric condition (mean ± standard deviation: 0.891 ± 0.081) are significantly higher (p<0.001) than these during the dynamic condition (mean ± standard deviation: 0.858 ± 0.065). The results in this subsection present the promising potential of the US ERC normalization as an alternative and commonly effective muscle fatigue indicator.

### 3.3. Implication of US Echogenicity as a Fatigue Indicator

**Figure 8:**
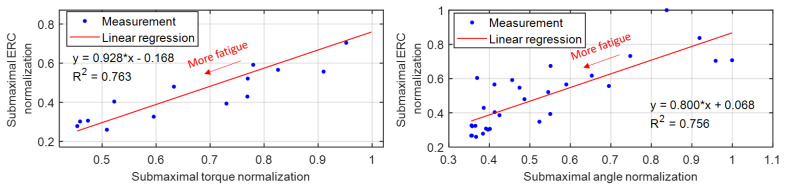
Linear relationships between the sub-maximal US ERC normalization and sub-maximal dorsiflexion torque/angle normalization under isometric/dynamic muscle fatigue progression conditions. Reported data are from Participant Sub05.

Figure 8 presents the representative scatter plots between the TA muscle’s sub-maximal US ERC normalization and the sub-maximal dorsiflexion torque normalization/angle normalization under isometric/dynamic fatigue progression conditions, where the data were collected from Participant Sub05. The direction of decreasing dorsiflexion torque or angle corresponds to the fatigue progression direction, as labeled in Figure 8. Through the linear regression model (the equations and $R^{2}$ values as shown in Figure 8), strong linear relationships between the sub-maximal US ERC and the sub-maximal dorsiflexion torque/angle were observed with the *p*-value of each slope from the *F*-statistic less than $10^{-4}$, which indicates that US ERC is a reliable alternative fatigue indicator for each participant. A summary of $R^{2}$, slope with *p*-value, and *y*-intercept with *p*-value from the linear regression analysis under isometric and dynamic fatigue progression conditions on all eight participants is given in Table 3. The results show that the mean slope values under isometric and dynamic conditions are both close to 1, while the mean y-intercept values are close to 0. Overall, the $R^{2}$ values are 0.840 ± 0.054 and 0.794 ± 0.065 under the isometric and dynamic conditions. The statistical analysis shows that the $R^{2}$ values under the isometric condition are significantly higher than those under the dynamic condition (*p*-value = 0.024). Therefore, the results imply that when using US ERC as the secondary fatigue indicator, the isometric scenario is likely to show significantly better fatigue-indicating performance than the dynamic scenario.

## 4. Discussion

The US echogenicity signal as an online FES-induced muscle fatigue indicator was investigated for the first time under the isometric and dynamic ankle dorsiflexion movements in this study. The experimental results on eight participants without any neurological disorders showed that the US ERC normalization was exponentially decreasing along with the muscle contraction cycles for both isometric ($R^{2}=0.891\pm 0.081$) and dynamic ($R^{2}=0.858\pm 0.065$) conditions. Additionally, the results also showed strong linear relationships between the US ERC normalization and dorsiflexion torque normalization ($R^{2}=0.840\pm 0.054$) or dorsiflexion angle normalization ($R^{2}=0.794\pm 0.065$) during the muscle fatigue progression. Interpretation of results, potential improvements, and applications will be discussed in the following parts.

In the experimental protocol, a zero-order-hold function was used to enable the data collection of the real-time US echogenicity signal at 1000 Hz. However, the US echogenicity update frequency was determined by the online imaging beamforming, processing, and gray-scaled analysis. In the current experimental setup and US imaging machine configurations, the online US echogenicity generation time was 127.9 ± 7.8 ms for a single image frame, which resulted in a US echogenicity updating frequency of 7.8 Hz. Compared to the US strain imaging computation time per image frame, 368.7 ± 7.2 ms [37], the computational load is significantly reduced by 65.3% (p<0.001) by using the US echogenicity as the FES-induced muscle fatigue indicator. Regarding the FES-induced muscle fatigue-indicating performance, the findings in [37] showed that under the isometric condition, the $R^{2}$ value of the linear regression model between sub-maximal mean (maximal) axial tissue strain normalization and sub-maximal joint torque normalization was 0.823±0.151 (0.850±0.165). A two-tail paired *t*-test did not show any significant difference between the $R^{2}$ values of the linear model by using US echogenicity and the $R^{2}$ values of the linear model by using US strain imaging. The advantages of using US echogenicity as a muscle fatigue indicator include (1) the relatively robust selection of the ROI due to the static nature, (2) no requirement of US image with higher resolution and clearly visualized architectural features, and (3) the significant reduction of calculation time for easier real-time implementation. Therefore, enough evidence implies that the US echogenicity has a comparable fatigue-indicating performance of FES-induced muscle fatigue as US strain imaging, but with a much lower computational intensity and a promising potential for online implementation for functional tasks, like drop-foot correction by using FES during walking.

The muscle force’s, joint torque’s or joint motion’s decay during the FES-elicited muscle contraction has always been taken as a gold standard indicator for peripheral muscle fatigue, but measures of muscle force, joint torque, or joint motion usually require sophisticated hardware setup and only provide mechanical-type signals without showing any neuromuscular changes during the muscle fatigue progression. In addition, switching between indicator platforms is required to evaluate muscle fatigue for both isometric and dynamic conditions. Therefore, introducing an alternative non-invasive FES-induced muscle fatigue indicator that can be easily implemented for both isometric and dynamic tasks, with a simpler setup and in a real-time manner, is necessary. The real-time US echogenicity measurement facilitates a simplistic evaluation of the current muscle fatigue levels so that users can adjust the corresponding stimulation intensity to increase the FES-related rehabilitative training period or terminate the rehabilitative training if the muscle is too fatigued. Furthermore, the US echogenicity-indicated muscle fatigue will also be beneficial to advanced closed-loop FES controller design with the consideration of muscle fatigue. The US echogenicity signal is potentially sensitive to several factors, including the elevation angle between the transducer arm and the skin surface, the orientation angle between the transducer array and the skin surface, the relative sliding between the transducer array and the skin surface, and the pressure on the skin. To mitigate these factors, a customized 3D-printed US transducer holder, detailed in [39,40], and elaborate experimental operations were utilized. First of all, the US transducer beam was tightly bonded onto the arm of the rotation component of the holder, which guaranteed the elevation angle to be approximately 90°, so the transducer was always perpendicular to the skin surface. Secondly, the US transducer was rotated to the cross-sectional direction to get a good view of the target TA muscle and then rotated to the longitudinal direction for real-time echogenicity data collection. Once the longitudinal direction was determined, no further rotation was conducted, so the orientation angle was set as the location where the transducer was at the longitudinal direction. Thirdly, Velcro straps were used to bond the base frame of the holder onto the skin tightly to avoid significant sliding of the US transducer, although there might be some squeezing of the TA muscle. Due to the compliant shape of the Velcro straps, when the TA muscle was bulging due to the stimulation, minimal transducer-to-skin pressure change was expected throughout each fatigue progression trial.

**Figure 9:**
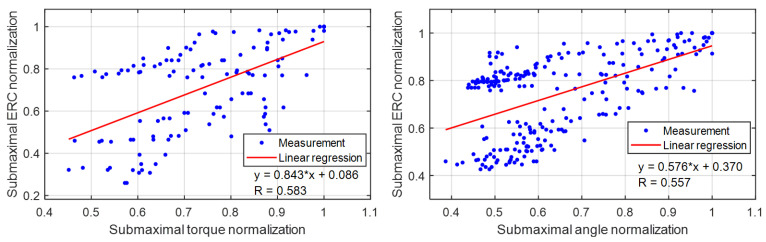
Summarized results of using US echogenicity as the FES-induced muscle fatigue indicator under both isometric and dynamic conditions. Reported data are from all eight participants.

To evaluate the generalization of using US echogenicity as an FES-induced muscle fatigue indicator, results from the individual participant as shown in Figure 8 are summarized in Figure 9. There are 120 data points (15 points from each participant × 8 participants) and 240 data points (30 points from each participant × 8 participants) for isometric and dynamic conditions, respectively. The linear regression equations and correlation coefficients are also labeled on the corresponding plots. From the F-statistic, the slope values for isometric and dynamic conditions are 0.843 and 0.576 with the *p*-values of $5.52^{-13}$ and $9.19^{-22}$, respectively, while the y-intercept values are 0.086 and 0.370 with the *p*-values of 0.38 and $1.76^{-19}$, respectively. It is observed that the correlation coefficient under the isometric condition is higher than that under the dynamic condition, which indicates the US echogenicity has a stronger correlation with the fatigue benchmark and potentially is a more accurate fatigue indicator when FES is applied under the isometric condition than the dynamic condition.

The results in Figure 9 showed a relatively high inter-subject variation of using US echogenicity as a muscle fatigue indicator under the application of FES in the current study. One possible reason is that the current work is a proof-of-concept study, which is not to develop a very generalized interface to predict FES-induced muscle fatigue. Instead, the purpose was to validate that the US echogenicity signal can be used as a personalized muscle fatigue indicator when FES is applied. The diversity most likely resulted from the personalized muscle contraction pattern and the personalized ultrasound echogenicity relative change during the FES-induced muscle fatigue protocol under both isometric and dynamic conditions. Furthermore, due to the variations of muscle size, recruitment pattern, FES electrode placement, and ultrasound transducer placement among different participants, the same submaximal dorsiflexion torque/angle change from different persons is likely to cause different submaximal ERC change. Another possible reason would be the relatively small population size in the current study, which will be further validated in a larger number of participants and multiple groups of different muscle conditions in future work. In addition, the findings in the current study indicate that the US echogenicity as an indicator of FES-induced muscle fatigue behaves better under the isometric condition than the dynamic condition. This observation corresponds to the results related to evoked EMG (eEMG) as an indicator [44], where the eEMG is effective at quantifying muscle force and fatigue during the isometric contraction but may not be effective during dynamic contractions including cycling and stepping. However, one limitation is that no muscle fatigue-indicating performance comparison between the use of US ERC and the use of sEMG during the same FES-induced muscle fatigue progression is presented in the current study. Inspired by the studies in [39,40,45], future work will investigate the FES-induce muscle fatigue indicators by using sole sEMG signal, sole US echogenicity signal, and the potential fusion of sEMG and US echogenicity signals.

## 5. Conclusions

In the current work, we investigated the use of temporal US echogenicity to quantitatively assess the muscle fatigue elicited by FES under both isometric and dynamic ankle dorsiflexion functionalities. The results showed that the US ERC expressed an exponential reduction along with the muscle contraction cycles both in isometric and dynamic conditions. Furthermore, the results of linear regression analysis showed strong linear relationships between the US ERC normalization and the gold standard fatigue indicators, namely, isometric dorsiflexion torque normalization or dynamic dorsiflexion angle normalization. The comparison between the current work and existing studies verified that the US ERC is a comparable fatigue indicator to axial tissue strain imaging during the isometric fatigue progression, but with a realistic computation time for real-time implementation. The findings in the current work indicate that the US echogenicity is a promising non-invasive and computationally efficient measure for assessing FES-induced muscle fatigue, and potentially, it can be integrated into an advanced FES controller design that considers muscle fatigue in real-time.

## Figures and Tables

**Table 1:** FES pulse width threshold and saturation values from each participant (Unit: μs).

Participant	Sub01	Sub02	Sub03	Sub04	Sub05	Sub06	Sub07	Sub08
Threshold	100	40	20	20	60	80	60	40
Saturation	420	580	520	500	520	500	400	560

**Table 2:** Coefficients of exponential regression models (y=aexp(bx)+c) and $R^{2}$ values between each variable and the TA muscle contraction cycles.

Participants	Coefficients and $R^{2}$ of Exponential Regression Models							
	Isometric Condition				Dynamic Condition			
	a	b	c	$R^{2}$	a	b	c	$R^{2}$
Sub01	0.955	−0.022	0.015	0.929	0.952	−0.016	0.115	0.923
Sub02	0.948	−0.018	0.098	0.919	0.600	−0.020	0.405	0.904
Sub03	0.931	−0.019	0.005	0.876	0.894	−0.034	0.186	0.965
Sub04	0.515	−0.020	0.502	0.942	0.732	−0.048	0.453	0.940
Sub05	0.616	−0.019	0.760	0.957	0.733	−0.037	0.377	0.926
Sub06	0.981	−0.011	0.428	0.803	0.478	−0.037	0.526	0.888
Sub07	0.824	−0.018	0.301	0.904	0.518	−0.049	0.567	0.911
Sub08	0.835	−0.031	0.165	0.925	0.457	−0.053	0.631	0.907
Sub01	0.581	−0.082	0.598	0.967	0.634	−0.025	0.436	0.919
Sub02	0.390	−0.036	0.193	0.772	0.751	−0.013	0.119	0.763
Sub03	0.643	−0.073	0.497	0.899	0.867	−0.062	0.350	0.857
Sub04	0.695	−0.060	0.452	0.966	0.622	−0.026	0.449	0.919
Sub05	0.730	−0.036	0.193	0.771	0.751	−0.013	0.119	0.763
Sub06	0.665	−0.055	0.455	0.966	0.642	−0.046	0.408	0.919
Sub07	0.618	−0.057	0.398	0.891	0.691	−0.038	0.309	0.863
Sub08	0.724	−0.046	0.344	0.895	0.685	−0.039	0.308	0.865

**Table 3:** Coefficients of linear regression models (y=ax+b) and $R^{2}$ values between dorsiflexion torque/angle normalization and ERC normalization.

Participants	Coefficients and $R^{2}$ of Linear Regression Models									
	Isometric Condition					Dynamic Condition				
	*a*	*p*-Value	*b*	*p*-Value	$R^{2}$	*a*	*p*-Value	*b*	*p*-Value	$R^{2}$
Sub01	0.895	$1.30e^{-6}$	0.245	0.091	0.889	1.009	$2.22e^{-14}$	−0.014	0.773	0.879
Sub02	0.894	$2.81e^{-5}$	−0.118	0.025	0.852	1.036	$1.92e^{-8}$	−0.155	0.079	0.682
Sub03	0.879	$3.44e^{-10}$	0.124	0.002	0.879	0.752	$6.55e^{-9}$	0.161	0.003	0.827
Sub04	1.475	$3.30e^{-8}$	−0.555	$1.17e^{-4}$	0.911	0.900	$6.14e^{-13}$	0.126	$5.90e^{-3}$	0.847
Sub05	0.928	$1.65e^{-5}$	−0.168	0.117	0.763	0.800	$5.80e^{-8}$	0.068	0.274	0.756
Sub06	0.754	$2.18e^{-5}$	0.206	0.224	0.843	1.245	$1.25e^{-12}$	−0.144	0.036	0.839
Sub07	1.319	$4.65e^{-6}$	−0.248	0.026	0.811	1.019	$1.35e^{-11}$	0.005	0.261	0.763
Sub08	1.169	$1.23e^{-5}$	−0.234	0.083	0.771	0.955	$1.34e^{-7}$	0.133	0.155	0.755
Mean	1.039	-	−0.094	-	0.840	0.965	-	0.023	-	0.794
Standard deviation	0.253	-	0.271	-	0.054	0.154	-	0.123	-	0.065

## Data Availability

All data in this study are available upon request to the correspondence author.

## Supplementary Materials

The following supporting information can be downloaded at: https://zenodo.org/record/5637439, Video S1: Dynamic_fatigue_Sub03; Video S2: Dynamic_fatigue_Sub05; Video S3: Isometric_fatigue_Sub03; Video S4: Isometric_fatigue_Sub05.

## Abbreviations

The following abbreviations are used in this manuscript:

SCI	spinal cord injury
CNS	central nervous system
FES	functional electrical stimulation
EMG	electromyography
sEMG	surface electromyography
eEMG	evoked electromyography
US	ultrasound
TA	tibialis anteior
ERC	echogenicity relative change
IRB	institutional Review Board
$R^{2}$	coefficient of determination
1D	one-dimensional
2D	two-dimensional
3D	three-dimensional
ROI	region of interest
RF	radio frequency

## References

[1] Zhang Q., Iyer A., Lambeth K., Kim K., Sharma N. Ultrasound Echogenicity-based Assessment of Muscle Fatigue During Functional Electrical Stimulation. Proceedings of the 2021 Annual International Conference of the IEEE Engineering in Medicine and Biology Society.

[2] The National SCI Statistical Center (2017). Spinal Cord Injury (SCI) Facts and Figures at a Glance.

[3] Virani S.S., Alonso A., Benjamin E.J., Bittencourt M.S., Callaway C.W., Carson A.P., Chamberlain A.M., Chang A.R., Cheng S., Delling F.N. (2020). Heart disease and stroke statistics—2020 update: A report from the American Heart Association. Circulation.

[4] Iezzoni L.I., McCarthy E.P., Davis R.B., Siebens H. (2001). Mobility difficulties are not only a problem of old age. J. Gen. Intern. Med..

[5] Kantrowitz A. (1960). Electronic physiologic aids. Report of the Maimonides Hospital.

[6] Liberson W., Holmquest H., Scot D., Dow M. (1961). Functional electrotherapy: Stimulation of the peroneal nerve synchronized with the swing phase of the gait of hemiplegic patients. Arch. Phys. Med.

[7] Granat M.H., Maxwell D.J., Ferguson A.C., Lees K.R., Barbenet J.C. (1996). Peroneal stimulator: Evaluation for the correction of spastic drop foot in hemiplegia. Arch. Phys. Med. Rehabil..

[8] Lyons G., Sinkjaer T., Burridge J., Wilcox D. (2002). A review of portable FES-based neural orthoses for the correction of drop foot. IEEE Trans. Neur. Syst. Rehab. Eng..

[9] Kottink A.I., Oostendorp L.J., Buurke J.H., Nene A.V., Hermens H.J., IJzerman M.J. (2004). The Orthotic Effect of Functional Electrical Stimulation on the Improvement of Walking in Stroke Patients with a Dropped Foot: A Systematic Review. Artif. Organs.

[10] Everaert D.G., Thompson A.K., Chong S.L., Stein R.B. (2010). Does Functional Electrical Stimulation for Foot Drop Strengthen Corticospinal Connections?. Neurorehabil. Neural Repair.

[11] Kluding P.M., Dunning K., O’Dell M.W., Wu S.S., Ginosian J., Feld J., McBride K. (2013). Foot Drop Stimulation Versus Ankle Foot Orthosis After Stroke. Stroke.

[12] Melo P., Silva M., Martins J., Newman D. (2015). Technical developments of functional electrical stimulation to correct drop foot: Sensing, actuation and control strategies. Clin. Biomech..

[13] Sharma N., Kirsch N.A., Alibeji N.A., Dixon W.E. (2017). A Non-Linear Control Method to Compensate for Muscle Fatigue during Neuromuscular Electrical Stimulation. Front. Robot. AI.

[14] Nguyen R., Masani K., Micera S., Morari M., Popovic M.R. (2011). Spatially distributed sequential stimulation reduces fatigue in paralyzed triceps surae muscles: A case study. Artif. Organs.

[15] Sayenko D.G., Nguyen R., Hirabayashi T., Popovic M.R., Masani K. (2015). Method to reduce muscle fatigue during transcutaneous neuromuscular electrical stimulation in major knee and ankle muscle groups. Neurorehabil. Neural Repair.

[16] Sayenko D.G., Nguyen R., Popovic M.R., Masani K. (2014). Reducing muscle fatigue during transcutaneous neuromuscular electrical stimulation by spatially and sequentially distributing electrical stimulation sources. Eur. J. Appl. Physiol..

[17] Downey R.J., Bellman M.J., Kawai H., Gregory C.M., Dixon W.E. (2014). Comparing the induced muscle fatigue between asynchronous and synchronous electrical stimulation in able-bodied and spinal cord injured populations. IEEE Trans. Neural Syst. Rehabil. Eng..

[18] Downey R.J., Cheng T.H., Bellman M.J., Dixon W.E. (2015). Closed-loop asynchronous neuromuscular electrical stimulation prolongs functional movements in the lower body. IEEE Trans. Neural Syst. Rehabil. Eng..

[19] Vøllestad N.K. (1997). Measurement of human muscle fatigue. J. Neurosci. Methods.

[20] Sadoyama T., Miyano H. (1981). Frequency analysis of surface EMG to evaluation of muscle fatigue. Eur. J. Appl. Physiol. Occup. Physiol..

[21] Cifrek M., Medved V., Tonković S., Ostojić S. (2009). Surface EMG based muscle fatigue evaluation in biomechanics. Clin. Biomech..

[22] Rogers D.R., MacIsaac D.T. (2011). EMG-based muscle fatigue assessment during dynamic contractions using principal component analysis. J. Electromyogr. Kinesiol..

[23] Ibitoye M.O., Hamzaid N.A., Zuniga J.M., Wahab A.K.A. (2014). Mechanomyography and muscle function assessment: A review of current state and prospects. Clin. Biomech..

[24] Yoshitake Y., Ue H., Miyazaki M., Moritani T. (2001). Assessment of lower-back muscle fatigue using electromyography, mechanomyography, and near-infrared spectroscopy. Eur. J. Appl. Physiol..

[25] Praagman M., Veeger H., Chadwick E., Colier W., Van Der Helm F. (2003). Muscle oxygen consumption, determined by NIRS, in relation to external force and EMG. J. Biomech..

[26] Scano A., Pirovano I., Manunza M., Spinelli L., Contini D., Torricelli A., Re R. (2020). Sustained fatigue assessment during isometric exercises with time-domain near infrared spectroscopy and surface electromyography signals. Biomed. Opt. Express.

[27] Dawson M.J., Gadian D., Wilkie D. (1978). Muscular fatigue investigated by phosphorus nuclear magnetic resonance. Nature.

[28] Zhang Q., Hayashibe M., Fraisse P., Guiraud D. (2011). FES-induced torque prediction with evoked EMG sensing for muscle fatigue tracking. IEEE/ASME Trans. Mechatron..

[29] Ambrosini E., Ferrante S., Schauer T., Klauer C., Gaffuri M., Ferrigno G., Pedrocchi A. (2014). A myocontrolled neuroprosthesis integrated with a passive exoskeleton to support upper limb activities. J. Electromyogr. Kinesiol..

[30] Pilkar R., Yarossi M., Ramanujam A., Rajagopalan V., Bayram M.B., Mitchell M., Canton S., Forrest G. (2016). Application of empirical mode decomposition combined with notch filtering for interpretation of surface electromyograms during functional electrical stimulation. IEEE Trans. Neural Syst. Rehabil. Eng..

[31] Mandrile F., Farina D., Pozzo M., Merletti R. (2003). Stimulation artifact in surface EMG signal: Effect of the stimulation waveform, detection system, and current amplitude using hybrid stimulation technique. IEEE Trans. Neural Syst. Rehabil. Eng..

[32] Li Z., Guiraud D., Andreu D., Benoussaad M., Fattal C., Hayashibe M. (2016). Real-time estimation of FES-induced joint torque with evoked EMG. J. Neuroeng. Rehabil..

[33] Crouch D.L., Pan L., Filer W., Stallings J.W., Huang H. (2018). Comparing Surface and Intramuscular Electromyography for Simultaneous and Proportional Control Based on a Musculoskeletal Model: A Pilot Study. IEEE Trans. Neural Syst. Rehabil. Eng..

[34] Shi J., Zheng Y.P., Chen X., Huang Q.H. (2007). Assessment of muscle fatigue using sonomyography: Muscle thickness change detected from ultrasound images. Med. Eng. Phys..

[35] Witte R.S., Kim K., Martin B.J., O’Donnell M. Effect of fatigue on muscle elasticity in the human forearm using ultrasound strain imaging. Proceedings of the 2006 International Conference of the IEEE Engineering in Medicine and Biology Society.

[36] Sheng Z., Sharma N., Kim K. (2019). Quantitative Assessment of Changes in Muscle Contractility Due to Fatigue During NMES: An Ultrasound Imaging Approach. IEEE Trans. Biomed. Eng..

[37] Sheng Z., Sharma N., Kim K. (2021). Ultra-High-Frame-Rate Ultrasound Monitoring of Muscle Contractility Changes Due to Neuromuscular Electrical Stimulation. Ann. Biomed. Eng..

[38] Sikdar S., Rangwala H., Eastlake E.B., Hunt I.A., Nelson A.J., Devanathan J., Shin A., Pancrazio J.J. (2013). Novel method for predicting dexterous individual finger movements by imaging muscle activity using a wearable ultrasonic system. IEEE Trans. Neural Syst. Rehabil. Eng..

[39] Zhang Q., Iyer A., Kim K., Sharma N. (2021). Evaluation of Non-invasive Ankle Joint Effort Prediction Methods for Use in Neurorehabilitation Using Electromyography and Ultrasound Imaging. IEEE Trans. Biomed. Eng..

[40] Zhang Q., Kim K., Sharma N. (2020). Prediction of Ankle Dorsiflexion Moment by Combined Ultrasound Sonography and Electromyography. IEEE Trans. Neural Syst. Rehabil. Eng..

[41] Kirsch N., Alibeji N., Sharma N. (2017). Nonlinear model predictive control of functional electrical stimulation. Control Eng. Pract..

[42] Seynnes O.R., Cronin N.J. (2020). Simple Muscle Architecture Analysis (SMA): An ImageJ macro tool to automate measurements in B-mode ultrasound scans. PLoS ONE.

[43] Gavin H.P. (2019). The Levenberg–Marquardt Algorithm for Nonlinear Least Squares Curve-Fitting Problems.

[44] Ibitoye M.O., Estigoni E.H., Hamzaid N.A., Wahab A.K.A., Davis G.M. (2014). The effectiveness of FES-evoked EMG potentials to assess muscle force and fatigue in individuals with spinal cord injury. Sensors.

[45] Zhang Q., Iyer A., Sun Z., Kim K., Sharma N. (2021). A Dual-modal Approach Using Electromyography and Sonomyography Improves Prediction of Dynamic Ankle Dorsiflexion Motion. IEEE Trans. Neural Syst. Rehabil. Eng..

